# An Observation-Driven Agent-Based Modeling and Analysis Framework for *C. elegans* Embryogenesis

**DOI:** 10.1371/journal.pone.0166551

**Published:** 2016-11-16

**Authors:** Zi Wang, Benjamin J. Ramsey, Dali Wang, Kwai Wong, Husheng Li, Eric Wang, Zhirong Bao

**Affiliations:** 1 Department of Electrical Engineering and Computer Science, University of Tennessee, Knoxville, Tennessee, United States of America; 2 Joint Institute for Computational Sciences, University of Tennessee, Knoxville, Tennessee, United States of America; 3 Climate Change Science Institute, Oak Ridge National Laboratory, Oak Ridge, Tennessee, United States of America; 4 Farragut High School, Knoxville, Tennessee, United States of America; 5 Developmental Biology Program, Sloan Kettering Institute, New York City, New York, United States of America; University of Colorado Boulder, UNITED STATES

## Abstract

With cutting-edge live microscopy and image analysis, biologists can now systematically track individual cells in complex tissues and quantify cellular behavior over extended time windows. Computational approaches that utilize the systematic and quantitative data are needed to understand how cells interact *in vivo* to give rise to the different cell types and 3D morphology of tissues. An agent-based, minimum descriptive modeling and analysis framework is presented in this paper to study *C. elegans* embryogenesis. The framework is designed to incorporate the large amounts of experimental observations on cellular behavior and reserve data structures/interfaces that allow regulatory mechanisms to be added as more insights are gained. Observed cellular behaviors are organized into lineage identity, timing and direction of cell division, and path of cell movement. The framework also includes global parameters such as the eggshell and a clock. Division and movement behaviors are driven by statistical models of the observations. Data structures/interfaces are reserved for gene list, cell-cell interaction, cell fate and landscape, and other global parameters until the descriptive model is replaced by a regulatory mechanism. This approach provides a framework to handle the ongoing experiments of single-cell analysis of complex tissues where mechanistic insights lag data collection and need to be validated on complex observations.

## Introduction

Recent breakthroughs in light microscopy have opened new doors to study complex tissues *in vivo* at single-cell resolution. With genetically encoded fluorophores, 3D time-lapse imaging has provided unprecedented temporal and spatial resolution to observe cellular dynamics in diverse organisms. The recordings often contain hundreds to thousands of cells over hours to days of development, with sub-cellular spatial resolution and second- to minute-level temporal resolution. Such images offer cellular level systems analysis. How to utilize the complex information, however, in the large image series still presents a significant challenge.

In organisms such as the Nematode *C. elegans*, which is amenable to large-scale imaging and genetic experiments, systematic single-cell analysis has led to the highly desired quantitative measurement of cellular behaviors and unparalleled opportunities for such studies [1, 2]. During the first few hours, the embryo undergoes several rounds of cell division to generate hundreds of cells. Based on 3D time-lapse imaging, the entire cell lineage can be automatically traced to this stage, with every cell tracked through its movements and cell division. Quantitative measurements can be made on every cell to characterize its developmental behavior [3–6]. Most recently, this has led to over 4,000 measurements per embryo in terms of tissue type, proliferation (cell cycle length) and migration [7]. Furthermore, lineage analysis has been combined with systematic genetic perturbation. A recent study analyzed the loss of function phenotypes of genes that are essential for embryogenesis and generated a multi-scale model that contains a number of cell fates and cell-specific gene networks that regulate cell fate differentiation [2, 8]. Clearly, computational synthesis is required to understand the developmental mechanisms revealed by such complex data and to generate testable hypotheses.

Agent-based modeling (ABM) [9] is a powerful approach to study systems consisting of self- and environment-ruling, interacting agents [10–13], and it is well suited for multi-scale analysis of complex tissues and development. A desired framework would include players at multiple scales, namely how genes interact to give rise to cellular behavior and how cells interact to give rise to an organism. An ABM framework for complex tissues typically includes three scales: molecules (genes), cells, and tissues, in which agents represent interacting individual cells. Behaviors of an agent are controlled by inherited biological information: known actions of gene; intercellular signals, physical limitations, environment factors and external interference; or statistical models based on experimental measurements if the underlying molecular mechanism is not known. Emergent properties, such as coordinated generation of the cell types and tissue morphology, can be examined by simulating gene actions and cell interaction. Among the earlier ABM-related approaches, the Cellular Potts Model (CPM) [14, 15] and statecharts [16–19] are two representative ones that are worth noting. These models effectively simulate certain kinds of cell behavior, such as morphology, tissue development, and organogenesis. Such models focus on examining known/prescribed mechanisms, and are not aimed at, or even capable of handling, large amounts of observational/phenomenological data from the live microscope and 3D time-lapse imaging.

These considerations lead to our design and implementation of an observation-driven ABM framework for *C. elegans* embryogenesis, in which related fundamental aspects are included, such as lineage identity, cell fate, cell division timing and direction, and cell movement pathway. The framework incorporates large amounts of observational/phenomenological data until they are replaced by regulatory mechanisms to deal with the scenario where regulatory mechanisms lag data collection and potential mechanistic insights need to be examined against complex phenomena. Consequently, what is achieved and validated in this paper is a simple/clean way to (1) capture observational data with statistic models and (2) generate system level results (lineage, morphology). The rest of this paper is organized as follows: a multi-scale modeling and analysis framework, based on agent-based modeling concepts, is first described in the next section. Then the framework is validated using the observational *C. elegans* datasets, and results are analyzed. At the end of the paper, further model enhancements and computational experiments are discussed.

## Methodology

In recent years, many platforms for agent-based modeling have emerged to achieve specific goals, such as NetLogo [20], FLAME [21], MASON [22], Repast [23], and others. NetLogo is used in this study because NetLogo is straightforward in implementing the agent-based modeling concept for cell development. With its own syntax and grammar, as well as a friendly graphical interface, NetLogo is very easy to use, meaning that we can setup, run, and observe models without writing codes with complicated programming languages. A number of built-in models related to biology can greatly help our current model. Moreover, NetLogo supports 3D modeling, which is appropriate for simulating morphogenesis of tissues. Also, the built-in visualization provides useful tools for model verification. NetLogo’s main problem is its lack of speed [24], but this is not an issue at current scope of the project. Therefore, NetLogo is an appropriate choice of platform. The ODD (overview, design concepts, and details) protocol [25] is used to describe our model below.

### Overview

The following sub-sections present a brief description of the framework, including the purpose of this simulation, key variables used for constructing the model, and the overall framework. Details are then provided in the “Detail” sub-section.

#### Purpose

The long-term goal of this study is to provide a general platform to incorporate experimental data at the molecular and cellular levels to examine emergent properties of *C. elegans* embryogenesis. The purpose of the current study is to provide a basic implementation that can be extended to incorporate complex experimental data. In this regard, the eggshell, body axes and the lineage (mother-daughter relationship) are implemented to provide the foundation to describe embryogenesis. The implementation of cellular behaviors is limited to cell division and cell migration, and each behavior is controlled by a statistical model (mean and standard deviation) instead of through gene networks. The goal is to provide timely validation of the overall design and implementation. Different tools are also implemented to plot and visualize the results of simulation and examine the key temporal and spatial features of the simulated embryo.

#### State variables and scales

In order to simulate cell behaviors during development, two kinds of parameters are defined in advance: (1) attributes of the agent (cells in the framework); and (2) environmental parameters, such as spatial and temporal scales. The variables used in each category and a brief description of each variable are provided in Tables 1 and 2, respectively.

**Table 1:** Agent variables used in model.

Variable Name	Brief Description
name	Used for keeping track of a cell when looking up division directions; grabbed when cell divides from division direction file.
cellSize	Size of the cell, may change as time goes by or through division.
divTime	Contains time at which the cell divides in seconds; calculated during the divide function.
stdDev	Standard deviation of cycle length; read in from given time table and used in calculating divTime.
divX, divY, divZ	Holds division direction unit vector; calculated during divide function.
divTick	Used to determine if the cell is in its division cycle or in normal migration.
xCor, yCor, zCor	Hold the current X, Y and Z coordinates of a cell.
cellMoveX, cellMoveY, cellMoveZ	Hold the X, Y and Z coordinates of the next place that the cell will move to.
cellTargetX, cellTargetY, cellTargetZ	Long term target of cells. Hold the X, Y and Z coordinates of the destination that the cell will move to.

**Table 2:** Environmental variables used in model.

Variable Name	Brief Description
divCycleTime	Division cycle time of a cell, the default value is 90 s.
outTimeResolution	Time interval for output information of cell’s status, the default value is 60 s.
inEllipsoid	Used to determine if the cell is in a limited space of environment.

#### Process overview and scheduling

Our ABM framework for *C. elegans* embryogenesis contains agents corresponding to the exact number of cells from the 4-cell to 350-cell stage, as well as the mother-daughter relationships between cells known as the cell lineage. The current implementation includes three behaviors for each cell: cell-cycle length, the direction in which the cell divides, and its migration paths. Each of these behaviors is modeled based on statistical measurements in the wild-type embryo [7]. The model also includes an ellipsoidal eggshell and three body axes of the embryo.

**Fig 1:**
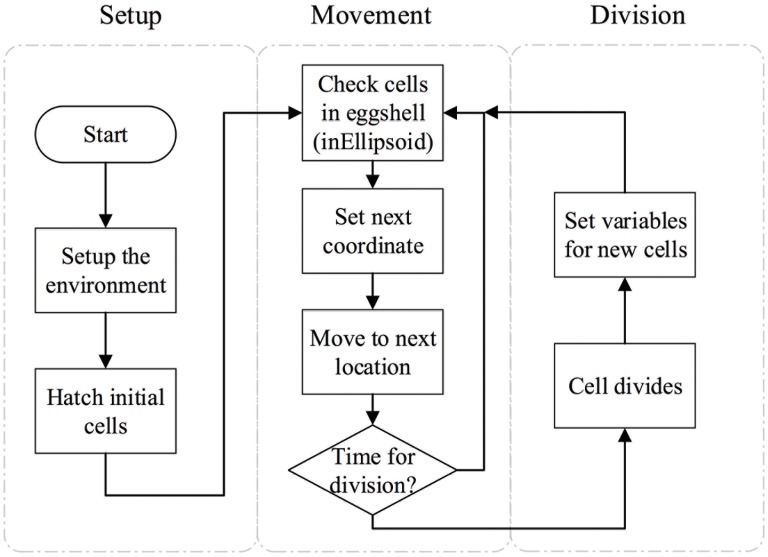
High level representation of framework. Two processes in the the framework, namely “setup” and “execution”. The “execution” process is further divided into a “movement” and a “division” activity.

On a higher level, our model can be divided into two processes (Fig 1): (1) the “setup” process and (2) the “execution” process (including a “movement” activity and a “division” activity). The setup process establishes the environment in which cells live and initializes a cell’s state such as nuclear size and coordinates. This will be further described in the “Initialization” section.

The “execution” phase is further divided into two biological stages, namely the interphase stages and mitosis for each cell cycle [26]. During the interphase, a cell moves in the ellipsoidal eggshell. The movement is controlled by several conditions of the environment and attributes of the cell itself. During mitosis, a cell divides. Two new cells are generated and initialized, replacing the old parent cell. The timing and direction of the division are controlled by attributes of the cell (see Table 1). As time goes by, the model continuously checks whether a cell is in the ellipsoidal eggshell, whether it is at the movement stage or enters the division stage, and implements the right actions. Detailed descriptions of each activity are provided in the “Sub-models” sub-section.

### Design concepts

A number of general concepts in ABM apply to this framework for *C. elegans* embryogenesis.

The fundamental goal of this agent-based *C. elegans* modeling framework includes understanding how cells become the appropriate cell type and find their appropriate positions through cell-cell interactions. The objectives of a cell include becoming the appropriate type and moving to the appropriate position based on the type and position of its neighbors. In the current implementation, a cell’s type is modeled as its lineal identity. In wild-type embryogenesis, a cell’s type equates with its lineal identity. At each time point, a cell adapts the direction of its movement based on its current position relative to the destination, moves to the next location, and continuously checks the division timing with appropriate stochasticity built into it. The model is designed to regularly output the agent’s status in standard data format. Such information is further used to validate the model by comparing it with observational data.

### Details

#### Initialization

The overall initiation of the simulation is achieved through a “setup” button in NetLogo. It triggers the import of various inputs that are derived from observational data (see the “Input and output” section) and sets the timer. Most specifically, four initial cells are hatched by the following procedures:

1. Set the name, size and original location (the X, Y, Z coordinates) for each cell.
2. Arrange a new color for every current cell in order to distinguish their later generations from other initial parents and their offspring.
3. Import divTime and the standard deviation.
4. Set the division direction (divX, divY, divZ) and make divTick temporarily unavailable.
5. Import the target destination (cellTargetX, cellTargetY, cellTargexZ) and calculate the next location (cellMoveX, cellMoveY, cellMoveZ) for current cell.
6. If there exist other cell(s) to be hatched, go to step 1.

#### Input and output

To achieve the goals of the cell development simulation, three groups of data are introduced at the current stage. The “timeTable” holds an index of all cells by their names, time points and durations of division, along with their standard deviations, respectively. A “dirTable” is introduced for keeping names of cells and their two daughters. Also, there are three other parameters recording the movement directions of the newly hatched cells. Finally, a “positionTable” is always necessary for maintaining every cell’s destination target.

In Table 3, a brief description and example of each parameter are given.

**Table 3:** Input parameters of each table and example values.

Parameters	Brief Description	Example
**timeTable**		
Tname	Names of every cell, appear to be an index.	“ABal”
divTime	Time point when the certain cell divide	41.8
divStdDev	Standard deviation of divTime, may be “-1” if it is not available.	0.6
cycleTime	Duration of division of certain cell.	14.8
cycleStdDev	Standard deviation of cycleTime, may be “-1” if it is not available.	0.6
**dirctionTable**		
parent	Names of every parent cell, appear to be an index.	“ABa”
daughter1	New generated cell.	“ABal”
daughter2	The other new generated cell, moves opposite to daughter1.	“ABar”
dirX, dirY, dirZ	3-D movement direction of daughter1.	(0.285, -0.547, 0.686)
**positionTable**		
Pname	Names of every cell, appear to be an index.	“ABal”
targetX, targetY, targetZ	Coordinate of certain cell’s destination.	(-10.371, 0.649, 2.536)

For the output, to establish a standard form for not only the current simulation, but further research as well, the data is organized as a format including 20 fields. In such format, mother-daughter relationship is coded in the first four columns: Column 1 represents an arbitrary index for a cell, specific to the time point. Column 2 is the index of the corresponding cell at the previous time point, either the cell itself or its mother, and −1 means NULL. Columns 3 and 4 are introduced as indices of the corresponding cell(s) at the next time point, either the cell itself or its two daughters. When the cell is not dividing, column four is set to −1. Columns 5 to 10 provide the x, y, z coordinates, diameter and name of a cell, respectively. Columns 11 to 20 are unused for current simulation, but will be used for further research. They are set to 0 except Column 15 is left blank.

An example of output data format is given in Table 4 for intuitive understanding.

**Table 4:** Example of output data format.

Variable Name	M-D relationship	x,y,z coordinates	diameter	Name	Column 11–20
Example	1, 1, 1, 1, -1	48, -121, 4	78	ABp	0, 0, 0, 0, 0, 0, 0, 0, 0

The parameter “outTimeResolution” (see Table 2) functions as controlling the output interval of the standard format data. In our model, the default value is 60 seconds.

#### Sub-models

As previously mentioned in the section “Process overview and scheduling” section, behaviors of the agents are divided into two activities: movement and division. Each is described in detail below.

**Fig 2:**
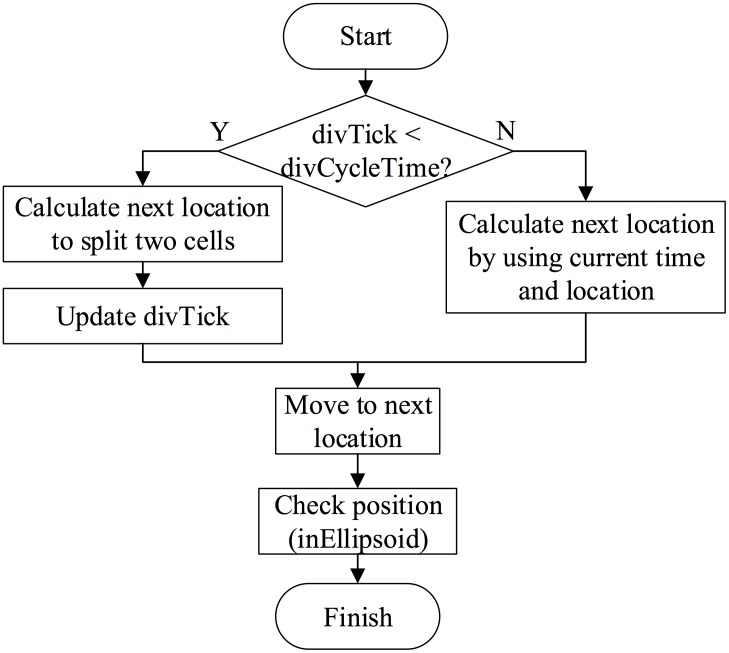
Movement activity of the model.

In the “movement” process, a cell moves to its target destination, where the next division takes place. To achieve this goal, two kinds of calculation of the cell’s location are implemented. (1) When a cell is just divided into two single ones, the primary thing is to split the two cells completely, without any overlap. In our model, we set the variable divCycleTime to 90 seconds for this process. (2) After that, the cell’s next location is calculated based on the differences between current time and cell’s division time (divTime), current location and its target destination. Eqs (1) and (2) are introduced to achieve such calculations Finally, we check whether each cell is still in the eggshell. The whole process of “movement” activity is shown in Fig 2.

cellMove=(cor+((div×cellSize/2)/(divCycleTime))), (1)

where **cellMove** = [*cellMoveX*, *cellMoveY*, *cellMoveZ*], **cor** = [*xcor*, *ycor*, *zcor*] represents the current coordinates of a cell, and **div** = [*divX*, *divY*, *divZ*].

cellMove=(cellTarget-cor)/(divTime-ticks), (2)

where **cellTarget** = [*cellTargetX*, *cellTargetY*, *cellTargetY*] and *ticks* represents the current time.

**Fig 3:**
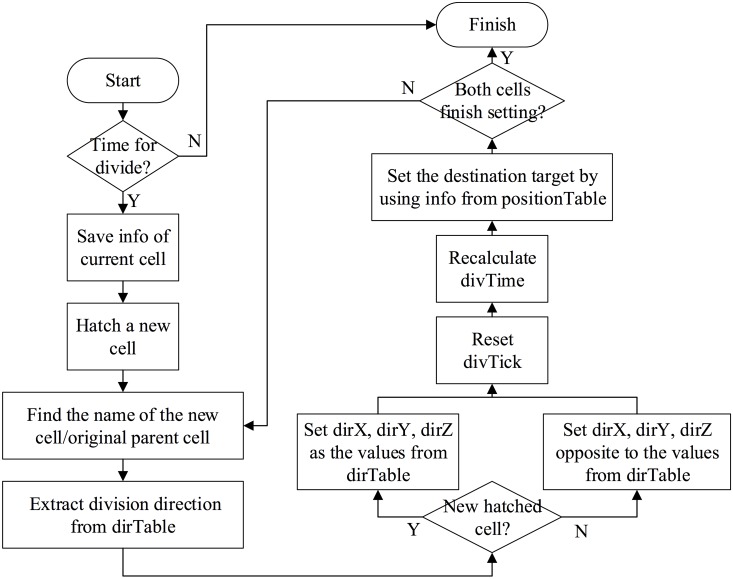
Divide activity of the model.

When it is time for division, information on the cell to be divided is temporarily saved for further potential usage. After that, two new daughter cells are generated, replacing the old parent one. In the current simulation, new cells are named by finding out their parents’ names in dirTable, and then obtaining the corresponding daughters’ names (see Table 3). Also, division directions are extracted from dirTable and transmitted into the cells’ attributes dirX, dirY and dirZ, which are used for the direction setting. DivTick, representing the current status of a cell, should be reset and goes into the next cycle. This operation helps to determine whether a cell is dividing, that is, separating into two halves, or has divided, and then to choose a corresponding method (see Eqs (1) and (2)) to calculate its next location in the “movement” activity. When this is done, information on division time point and duration from the timeTable is extracted, and a new divTime for cells are recalculated by adding random disturbances based on their standard deviations. Finally, destination target coordinates for the cell are obtained by indexing its name in positionTable. The whole process is shown in Fig 3.

## Results and Discussion

### Model validation strategies

The long-term objectives and potential research implications of this project aim to allow biologists to generate testable hypotheses about cell development. The short term objectives are complete verification, to confirm that the framework is properly built.

We use a MATLAB application to verify the division cycle movement, by comparing the standard format output simulation data with the observational data. The MATLAB application used is named Dev-scape, which is an open source software that was first presented in [7]. The software package provides a comprehensive analysis of a cell’s differentiation, proliferation and morphogenesis at single-cell resolution. Input for Dev-scape is exactly the standard format output file of NetLogo. All the text or graphic information for validation, such as cell cycle, division time, and others, will be outputted for further analysis. Besides Dev-scape, we also use Java for the visualization of cell lineage tree, and POV-Ray [27] to analysis the cell migration process. Inputs for the above two validations are also obtained from Dev-scape.

Specifically, two main points of verification are introduced for the current stage of simulation, division cycle movement, and cell migration.

### Computational experiments

Quantifying division timing is relatively simple, since every sixty seconds, the program outputs standard format data, from which the number of cells at current time point can be counted. A curve that presents the number of cells over time is created as we continuously track this value. Theoretically, the number of cells is rigorously limited by the division time and its standard deviation in the framework. As a result of this assertion, simulation results outputted from simulation should not show a large difference as compared with observational data.

#### Number of cells in embryo overtime is properly modeled

**Fig 4:**
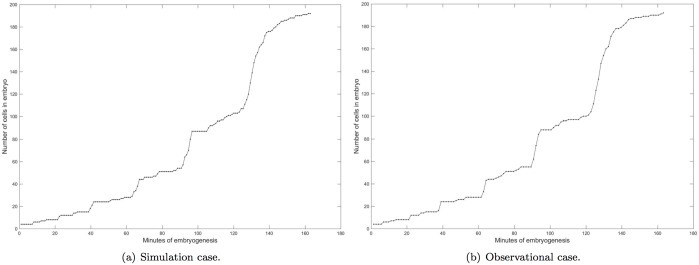
Number of cells in embryo over time. Figure shows the number of cells in the simulation/real embryo from the beginning to ∼180 minutes, from 4-cell stage to ∼180-cell stage.

From Fig 4 we can see that the two curves, representing the number of cells in the embryo from simulation and observation, respectively, largely overlap with each other, except that a few intervals have some slight deviation. This makes sense since for better recurring realistic situations, we add a random difference to the division time of each cell based on their statistical standard deviations.

Systematic and quantitative analysis of the result is implemented by utilizing root-mean-square error (RMSE) to measure the deviation between simulation and observational data (Eq (3)).

$$\sigma =\sqrt{\frac{\sum d_{i}^{2}}{n-1}},i=1,2,3,...,n,$$ (3)

where *n* is the number of measurements, and *d*_*i*_ is the deviation between simulation and observational data.

The number of cells in an embryo over time is sampled every 5 minutes from both simulation result and observational data. If data is missing at the minute multiple of 5, the nearest minute with data for both cases is chosen as the sampling point. Because of the frequent divisions of cells during some time intervals (especially 100 to 140 min), and the limited output resolution (60 s in this case), when the above two sampling rules cannot be met, we select the point with minimum time difference between simulation result and observation data instead of an approximate estimation (see Table 5).

**Table 5:** Number of cells in embryo over time sampled every 5 minutes from simulation and observational data.

Sampling point	Simulation result	Observational result	Deviation	Sampling point	Simulation result	Observational result	Deviation
**5**	4	4	0	**85**	51	55	4
**11**	6	7	1	**89**	54	55	1
**16**	8	8	0	**97**	87	88	1
**20**	8	8	0	**100**	87	88	1
**25**	12	12	0	**104**	87	92	5
**31**	14	15	1	**112**	96	97	1
**35**	15	15	0	**115**	99	97	2
**41**	24	24	0	**120**	103	100	3
**45**	24	24	0	124*	107	111	4
**49**	24	26	2	132**	154	162	8
**55**	26	28	2	140***	176	179	3
**60**	28	28	0	146****	185	187	2
**68**	44	44	0	152*****	188	189	1
**76**	47	51	4	**157**	190	190	0
**81**	51	52	1	**160**	191	190	1

The following sampling points for simulation result and observational data vary: *124.2464 and 124.2027, **132.3937 and 132.576, ***140.541 and 140.9492, ****146.6515 and 146.5313, *****152.762 and 152.1134.

From the result we find that the deviations increase when the number of cells increases rapidly, especially the interval between 100 and 140 minutes. For the other time intervals, the deviations are relatively small. With Eq (3), we calculate that the RMSE *σ* for this case is 2.491. The immediate follow-up efforts are to (1) optimize the “movement” and “division” function to reduce the RMSE *σ*, and (2) substitute statistical models with regulatory mechanisms to approach the RMSE we obtain here.

#### Simulation generated cell lineage tree and cell cycle length of founder cells during *C. elegans* early embryogenesis show consistencies with those from wild-type embryo

**Fig 5:**
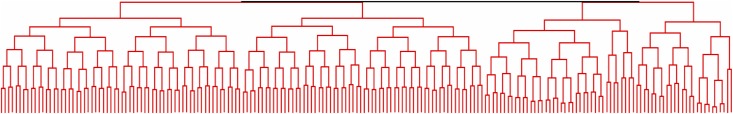
Cell lineage tree of simulation. Nodes represent cells with lines connecting mother and daughter cells. Length of the line represents cell cycle time.

A cell lineage tree drawn from simulation data is also provided (see Fig 5). The shape of this lineage tree intuitively describes every cell’s division timing, as well as different paces of cell divisions in their sublineages. We compare the simulation result with the cell lineage tree of wild-type embryos [28], and the large extent of similarity validates that division timing of each cell in simulation conforms to the situation of wild-type embryos. Also, consistency in the shape of both trees,where two sister cells show different paces of cell divisions in their sublineages, validates the correctness of body axes and cell naming.

Twelve founder cells in early embryogenesis are selected for the analysis of cell cycle length. We collect the born and divided times for each founder cell during simulation and compare the result with observational data (see Table 6). Cell cycle standard deviation is calculated based on born time and divided time standard deviations with Eq (4).

$$\sigma_{c}=\sqrt{\sigma_{b}^{2}+\sigma_{d}^{2}},$$ (4)

where *σ*_*c*_, *σ*_*b*_ and *σ*_*d*_ are the cell cycle, born time and divided time starnard deviations, respectively.

**Table 6:** Cell cycle analysis of 12 founder cells.

	Simulation result			Observational data						
Cell name	Born time	Divided time	Cell cycle	Born time	BTSD*	Divided time	DTSD**	Cell cycle	CCSD***	Deviation
**ABala**	2504	3604	1100	2508	36	3546	102	1038	108.1665	62
**ABalp**	2504	3503	999	2508	36	3540	102	1032	108.1665	33
**ABara**	2488	3498	1010	2514	36	3546	102	1032	108.1665	22
**ABarp**	2488	3492	1004	2514	36	3540	90	1026	96.933	22
**ABpla**	2522	3578	1056	2514	48	3522	96	1008	107.3313	48
**ABplp**	2522	3582	1060	2514	48	3552	96	1038	107.3313	22
**ABpra**	2501	3534	1033	2520	42	3540	96	1020	104.7855	13
**ABprp**	2501	3627	1126	2520	42	3582	108	1062	115.8792	64
**MS**	1860	2979	1119	1860	36	2928	84	1068	91.3893	51
**E**	1860	2948	1088	1860	36	2994	84	1134	91.3893	46
**C**	2094	3241	1147	2094	48	3198	108	1104	118.1863	43
**P3**	2094	3736	1642	2094	48	3630	132	1536	140.4564	106

*Born time standard deviation.

**Divided time standard deviation.

***Cell cycle standard deviation.

We find that most of the deviations of cell cycle length (11 of 12) between simulation result and observational data are less than 60% of the corresponding standard deviations. Moreover, the RMSE *σ* for cell cycle length is 52.877.

#### Cell migration patterns and distribution of positions

**Fig 6:**
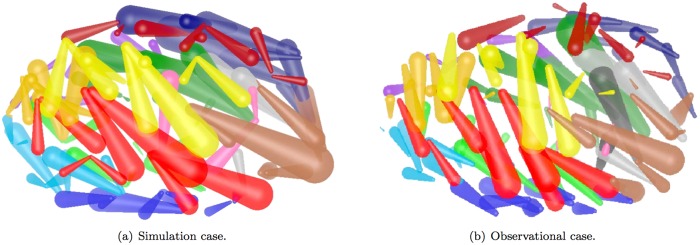
Cell displacement patterns during AB64 stage. 3D depth trajectories represent the movement path of cells. Different colors represent the 14 founder cells of *C. elegans*, which is color coded by the following rules: ABala(light brown), ABalp(light blue), ABara(light purple), ABarp(yellow), ABpla(red), ABplp(dark blue), ABpra(magenta), ABprp(dark purple), MSa(light green), MSp(dark green), E(pink), C(dark brown), D(light gray), P(dark gray).

Fig 6 demonstrates the cell migration of simulation and observational data, respectively, in which different types of cells are color coded. A 3D view of cell migration dynamics is provided during AB64 (when AB divides to 64 cells) stage. We cannot expect the two figures are completely the same, since the observational result is an average movement from large amounts of data, and on the other hand, random deviations (see Table 3 and the “Sub-models” section) are also added into the simulation model. The conclusion that trends of cell movement in simulation largely follows the situation in observation illustrates that the experimental result of cell displacement patterns looks good.

**Fig 7:**
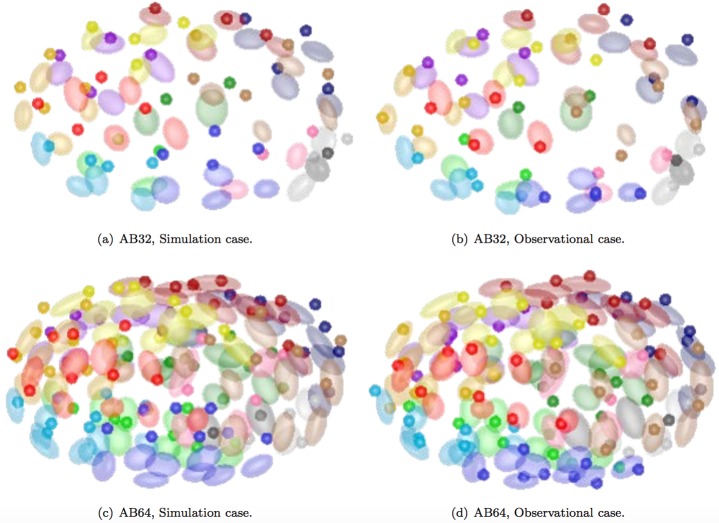
Cell position validation. Stages of AB32 and AB64 mean when the AB lineage divides into 32 and 64 cells. Color-coded rule is the same as in Fig 6. Dots represent the exact cell positions in a specific simulation/observational case, and ellipses represent the average position of each coresponding cell (with one standard deviation).

In Fig 7, we also visualize distributions of cell positions at AB32 and AB64 stage, respectively. Ellipsoids are drawn relying on a large amounts of data, centered at the average position of cell positions at the certain stage with radii of one standard deviation on each direction. The single point in each figure represents one certain result of cell position from simulation and observation. By comparing the corresponding figures in each stage for a number times of experiments, we observe that cells always locate near the spots where they are most likely to appear (in the ellipsoid), which is reasonable, even though there are slight differences between corresponding cells. Moreover, we examine whether the cumulative effect of positional deviations will have an influence on the results, and it turns out that errors produced at earlier stages are controlled in a proper range as they accumulate towards later stages.

Finally, we compare the positions of twelve founder cells at the time when they divide in simulation and observational cases (see Table 7). Deviations in each direction are produced owning the random errors added to the division times of all the cells. Compared with the standard deviations of observational data on each direction (data not shown here), the deviations between simulation result and observational data are extremely small. The RMSE on each direction *σ*_*x*_, *σ*_*y*_ and *σ*_*z*_ is 0.0059, 0.0067, and 0.0033, respectively.

**Table 7:** Analysis of dividing positions of 12 founder cells.

	Simulation result			Observational data			Deviation		
Cell name	x	y	z	x	y	z	x	y	z
**ABala**	-10.3181	-1.4295	2.156	-10.3185	-1.4285	2.1545	0.0004	0.001	0.0015
**ABalp**	-5.4404	6.4491	0.0724	-5.4435	6.4475	0.074	0.0031	0.0016	0.0016
**ABara**	-4.5968	-3.0929	4.6138	-4.596	-3.094	4.613	0.0008	0.0011	0.0008
**ABarp**	-0.6491	-5.1982	-2.2607	-0.649	-5.1985	-2.2605	0.0001	0.0003	0.0002
**ABpla**	-7.3182	-1.9064	-3.9134	-7.3145	-1.9025	-3.9135	0.0037	0.0039	0.0001
**ABplp**	1.2492	4.8501	-3.5957	1.2485	4.85	-3.5955	0.0007	0.0001	0.0002
**ABpra**	3.2836	-5.9391	2.2794	3.2845	-5.939	2.2795	0.0009	0.0001	0.0001
**ABprp**	8.8367	0.8474	2.6359	8.836	0.8445	2.636	0.0007	0.0029	0.0001
**MS**	-0.0008	0.004	-0.0022	0	0	0	0.0008	0.004	0.0022
**E**	9.1584	3.5893	-0.31	9.1555	3.5895	-0.3095	0.0029	0.0002	0.0005
**C**	5.1186	-2.3211	-3.7254	5.1119	-2.321	-3.725	0.0004	0.0001	0.0004
**P3**	12.252	3.723	0.1905	12.252	3.723	0.1905	0	0	0

## Conclusion and future work

In this paper, an agent-based *C. elegans* model has been developed to incorporate observational data on cellular behavior, such as cell lineage, fate, division time and direction, and movement path. Observation-driven data structures and flowcharts have been implemented in the framework to establish a statistic model, and regulatory mechanisms are allowed to be incorporated when more insights are discovered. System-level results have been generated, analyzed and validated in a simple and clean ways to gain potential possibilities to guide further *C. elegans* embryogenesis research. The software system is available through a public code repository at https://bitbucket.org/abm_utk/.

Our model considers fundamental aspects of *C.elegans* embryogenesis a whole process, rather than in separate modules [29]. This approach/design provides a framework to deal with experiments of complex tissues at a single-cell resolution under the circumstance that mechanistic rules lag data collection and remain to be substantiated on complex observations, which is of general interest.

Although we have built a basic descriptive agent-based model, and acquired a number of meaningful simulation results, a comprehensive framework including other main aspects during *C. elegans* embryogenesis is needed for further research and experimentation. Therefore, we design and reserve the following necessary interfaces, which can be considered as our immediate follow-up efforts.

### Gene list

Each agent should hold a list that contains genes related to the current model, with their genetic states and active states. Genetic information, as the basis of all agent (cell in our case) behaviors, is indispensable in agent-based *C. elegans* embryogenesis framework. Such kinds of structures built in the agents is also a prerequisite for further investigation of cell movement, division, polarity, interaction, fate, and landscape.

### Polarity and cell interaction

Polarity and cell interaction are the two most important ways to change genetic and active states of genes in cells. Such processes play significant roles during cell differentiation and control cell fate differentiation, and further impact other downstream cell activities, such as movement and division. Therefore, we maintain interfaces for these two processes, in which cells continuously seek the changes produced in themselves, from other cells, or the surrounding environment. When regulatory mechanisms that can lead to genetic change are targeted, states of the corresponding genes in certain target cells will change. The domino effect of the agent-based changes of gene states in huge network will provide us with much more interesting results than ever before.

### Cell fate and developmental landscape

As mentioned earlier, cell fate and developmental landscape [2] play important roles for research on developmental problems. The classic definition of cell fate is implemented by obtaining gene expression information in each cell. However, the limitation of such an approach is that it is quite difficult to acquire such a large amounts of data. Therefore, we reserve a hybrid definition of cell fate, in which the gene expression information in the cells themselves is not only utilized, but in their descendants is gathered as well. This allows us to maximize the potential to find more meaningful results by utilizing known knowledge obtained from regulatory mechanisms and experimentations.

### Global parameters

Factors that are not related to agent (cell) can be considered global parameters. It is obvious that such kind of factors will have a influence on biological processes. Interfaces reserved for this purpose will help us investigate the changes resulting from non agent-related factors: whether changes of temperature and humidity will result in different cell behaviors or fate changes during embryogenesis.

## Supporting Information

S1 File The initial file that contains the information of 4 founder cells. (TXT)

S2 File Information of the division time, cell cycle and their standard deviations of each cell. (TXT)

S3 File Cell position information. (TXT)

S4 File Information of the division direction, and the names of the daughters of each cell. (TXT)

S1 Dataset Output dataset. The data obtained from our NetLogo code, including the index, name, diameter, position, and mother-daughter relationship of each cell.
(ZIP)
